# Treatment of severe COVID-19 with human umbilical cord mesenchymal stem cells

**DOI:** 10.1186/s13287-020-01875-5

**Published:** 2020-08-18

**Authors:** Lei Shu, Changming Niu, Ruyou Li, Tingrong Huang, Yan Wang, Mao Huang, Ningfei Ji, You Zheng, Xiaolin Chen, Lei Shi, Mingjing Wu, Kaili Deng, Jing Wei, Xueli Wang, Yang Cao, Jiaxin Yan, Ganzhu Feng

**Affiliations:** 1 Department of Respiratory Medicine, Sir Run Run Hospital, Nanjing Medical University, Nanjing, 211166 Jiangsu China; 2 Department of Respiratory Medicine, the Second Clinical Medical School of Nanjing Medical University, Nanjing, 210011 Jiangsu China; 3 Department of Critical Care Medicine, the Second Affiliated Hospital of Nanjing Medical University, Nanjing, 210011 Jiangsu China; 4 Department of Respiratory Medicine, Huangshi Central Hospital, Hangshi, 435000 Hubei China; 5 Department of Nephrology, Huangshi Hospital of Traditional Chinese Medicine, Hangshi, 435000 Hubei China; 6 Jiangsu Cell Tech Medical Research Institute, Nanjing, 211166 Jiangsu China; 7 Department of Respiratory and Critical Care Medicine, the First Affiliated Hospital of Nanjing Medical University, Nanjing, 210029 China; 8 Department of Pulmonary and Critical Care Medicine, the Second Affiliated Hospital of Nanjing Medical University, Address: No. 121 Jiangjiayuan Rd, Gulou District, Nanjing, 210011 Jiangsu China

**Keywords:** Coronavirus disease-19 (COVID-19), Clinical characteristics, Coronavirus pneumonia, Human umbilical cord mesenchymal stem cells

## Abstract

**Background:**

COVID-19 is a highly infectious respiratory disease. No therapeutics have yet been proven effective for treating severe COVID-19.

**Objectives:**

To determine whether human umbilical cord mesenchymal stem cell infusion may be effective and safe for the treatment of severe COVID-19.

**Methods:**

Patients with severe COVID-19 were randomly divided into 2 groups: the standard treatment group and the standard treatment plus hUC-MSC infusion group. The incidence of progression from severe to critical illness, 28-day mortality, clinical symptom improvement, time to clinical symptom improvement, hematologic indicators including C-reactive protein, lymphocyte number, and interleukin 6, and imaging changes were observed and compared between the two groups.

**Measurements and main results:**

The incidence of progression from severe to critical illness and the 28-day mortality rate were 0 in the hUC-MSC treatment group, while 4 patients in the control group deteriorated to critical condition and received invasive ventilation; 3 of them died, and the 28-day mortality rate was 10.34%. In the hUC-MSC treatment group, the time to clinical improvement was shorter than that in the control group. Clinical symptoms of weakness and fatigue, shortness of breath, and low oxygen saturation obviously improved beginning on the third day of stem cell infusion and reached a significant difference on day 7. CRP and IL-6 levels were significantly lower from day 3 of infusion, the time for the lymphocyte count to return to the normal range was significantly faster, and lung inflammation absorption was significantly shorter on CT imaging in the hUC-MSC group than in the control group.

**Conclusions:**

Intravenous transplantation of hUC-MSCs is a safe and effective method that can be considered a salvage and priority treatment option for severe COVID-19.

**Trial registration:**

Chinese Clinical Trial Registration; ChiCTR2000031494; Registered on 2 April 2020; http://www.medresman.org

## Introduction

In December 2019, a series of pneumonia cases occurred in Wuhan City, Hubei Province, and other parts of China [1]. Recently, the causative agent was identified as a novel coronavirus, designated SARS-CoV-2 [2, 3], and this type of pneumonia was named coronavirus disease 2019 (COVID-19). Since then, the number of COVID-19 patients has sharply increased not only in China but also in most of the world. By April 5, 2020, there were more than 1,000,000 confirmed COVID-19 patients and more than 57,000 deaths in 207 countries, areas, or territories all over the world [4]. According to data from the WHO, the mortality of COVID-19 is 5.17%; in some places, however, the mortality rate is higher, reaching 16.7% [5], which depends on the sample size included and the severity of the outbreak. Therefore, controlling the mortality rate of critically ill patients is of paramount importance. Considering its high prevalence and infection rates, the World Health Organization (WHO) has declared COVID-19 a pandemic [6], and it has grown to be a public health emergency of international concern that represents an enormous threat to global health. However, thus far, no specific drugs or vaccines are available to treat COVID-19 patients.

Recently, studies have found that SARS-COV-2 interacts with human mucosal epithelial cells through ACE2 in a manner dependent on both binding of the viral spike (S) proteins to cellular receptors and S protein priming by host cell proteases [7, 8], which reveals cellular factors that may be used as therapeutic targets for controlling SARS-CoV-2 transmission.

Mesenchymal stem cells (MSCs) have been widely used in the clinical setting, not only for autoimmune diseases [9, 10] but also for infectious diseases [11–13], and their safety and effectiveness have been well elucidated. Umbilical cord mesenchymal stem cells (hUC-MSCs), a kind of MSC, can be easily obtained and expanded in vitro.

Numerous studies have shown that hUC-MSCs have significant immune modulation and tissue repair functions due to their low immunogenicity [14–16]. As an ideal candidate for allogenic adoptive transfer therapy, hUC-MSCs have been shown to play a protective role in A/H5N1-associated acute lung injury [17]. Although one article has been published recently on the therapeutic effects of stem cells on COVID-19, it was about bone marrow mesenchymal stem cells [18] and did not focus on the treatment of severe COVID-19. To date, the safety and therapeutic effect of hUC-MSCs on severe COVID-19 have not been reported.

To evaluate the efficacy of hUC-MSCs for treating severe COVID-19, we conducted this hUC-MSC transplantation pilot study to elucidate the potential therapeutic role of these cells in the treatment of severe COVID-19.

## Methods

### Study design and participants

This study was a single-center open-label, individually randomized, standard treatment-controlled trial conducted at Huangshi Hospital of Traditional Chinese Medicine in Hubei Province from Feb 12 to March 25, 2020, and it was performed according to the Declaration of Helsinki and approved by the Ethics Committee of the Huangshi Hospital of Traditional Chinese Medicine (No. HSZYPJ-2020-009-01). Written informed consent was obtained from all patients or their representatives when data were collected retrospectively.

The diagnosis of COVID-19 was based on WHO interim guidance [19] and a new coronavirus pneumonia diagnosis and treatment program (6th ed.) (in Chinese) [20]; the criteria for severe disease are (A) an epidemiological history; (B) etiological evidence (i.e., a positive SARS-CoV-2 nucleic acid test by the real-time reverse transcription polymerase chain reaction (RT-PCR) assay for SARS-CoV-2 RNA from the Chinese Center for Disease Control and Prevention following the protocol described previously [11, 21]); and (C) CT imaging indicators of pneumonia. In addition, these factors should coincide with any of the following criteria: (a) respiratory distress, respiration rate (RR) ≥ 30 times/min; (b) oxygen saturation ≤ 93% in the resting state; and (c) PaO_2_/FiO_2_ ≤ 300 mmHg (1 mmHg = 0.133 kPa). In general, patients with severe COVID-19 whose clinical symptoms were not significantly alleviated under standard treatment for 7 to 10 days were recommended for participation in this pilot study. The patients were randomly divided into 2 groups: a standard treatment group (control group) and a standard treatment plus umbilical cord mesenchymal stem cell infusion group (hUC-MSC group). The standard treatment was as follows: (1) supplemental oxygen (noninvasive or invasive ventilation), (2) antiviral agents (abidor/oseltamivir), (3) antibiotic agents (moxifloxacin is taken orally; if there is clear evidence of bacteriological infection, the choice of antibacterial drugs is based on a drug sensitivity test), and (4) glucocorticoid therapy (1–2 mg/kg, less than a week). Exclusion criteria included the following: any kind of cancer, severe liver disease, known allergy or hypersensitivity to hUC-MSCs, and other conditions that the clinician deemed inappropriate for participation.

### Cell preparation and transplantation

Clinical-grade hUC-MSCs were supplied, free of charge, by The Jiangsu Cell Tech Medical Research Institute and The Jiangsu Cell Tech Biotechnology Co. The product was registered and reviewed by the China Clinical Trial Center (Registration No. ChiCTR2000031494). It received approval from the Ethics Committee of Huangshi Hospital of Traditional Chinese Medicine (Approval Letter No. HSZYPJ-2020-009-01). Preparation was completed in a GMP laboratory. Cells at passages P3 to P5 were used and had the ISCT-recommended cell surface characteristics of MSCs, including expression (> 95%) of CD73, CD90, and CD105 and lack of cell surface presentation (< 2%) of CD34, CD45, CD14 or CD11b, CD79α or CD19, and HLA-DR [22, 23]. Intravenous administration was used. Before the intravenous drip, the MSCs were suspended in 100 ml of normal saline, and the total number of transplanted cells was calculated as 2 × 10^6^ cells/kg. The infusion was from the patients’ right cubital veins and lasted approximately 1 h (35 drops/min).

### Chest CT scans and imaging evaluation

All patients underwent chest CT scans from pretreatment to the follow-up period, and the characteristics of ground-glass opacity (GGO), consolidation, nodules, reticulation, interlobular septal thickening, crazy-paving pattern, linear opacities, subpleural curvilinear line, bronchial wall thickening, lymph node enlargement, pleural effusion, and pericardial effusion were evaluated based on the Fleischner Society Nomenclature recommendations and similar studies [24, 25]. To quantify the extent of the lesions, a thin-section CT score was used. Each lobe was assigned a score as follows: 0, 0% involvement; 1, less than 5% involvement; 2, 5 to 25% involvement; 3, 26 to 49% involvement; 4, 50 to 75% involvement; and 5, greater than 75% involvement. A score of 0–5 was assigned to each lobe, with a total possible score of 0–25 [25, 26]. The software Pulmonary Infection Assistant Diagnosis (V1.7.0.1, Dexin Medical Imaging Technology Co., Ltd., Xian City, Shaanxi Province, China) was applied for imaging evaluation.

### Clinical outcome assessment

The patients were observed for 2 weeks after hUC-MSC infusion, and clinical symptoms, laboratory tests, and radiological results were recorded and confirmed by experienced physicians. The primary clinical outcomes included the incidence of progression from severe to critical illness and the time to a clinical improvement of two points on a seven-category ordinal scale that has been used widely in clinical symptom assessment or discharge from the hospital [27]. In our study, the NEWS2 score and seven-category ordinal scale were used to assess the clinical symptoms and conditions of the enrolled patients [28]. The secondary clinical outcomes included patient status at days 7 and 14 assessed with a seven-category ordinal scale, hospital stay, changes in oxygenation index, hematological inflammatory factors, and imaging.

### Statistical analysis

Continuous variables with a normal distribution are expressed as the mean ± standard deviation (SD); nonnormally distributed continuous variables are reported as the median (interquartile range, IQR). For the *P* value, the Mann-Whitney *U* test was used to analyze normally distributed continuous variables, and the Kruskal-Wallis test was used for nonnormally distributed data. Categorical variables were presented as percentages and analyzed by the chi-square test or Wilcoxon rank-sum test. All statistical analyses were performed with Stata version 14.2 for Mac (StataCorp, College Station, TX), and a *P* value less than 0.05 was considered statistically significant.

## Results

### hUC-MSC treatment procedure and general patient information

**Fig. 1:**
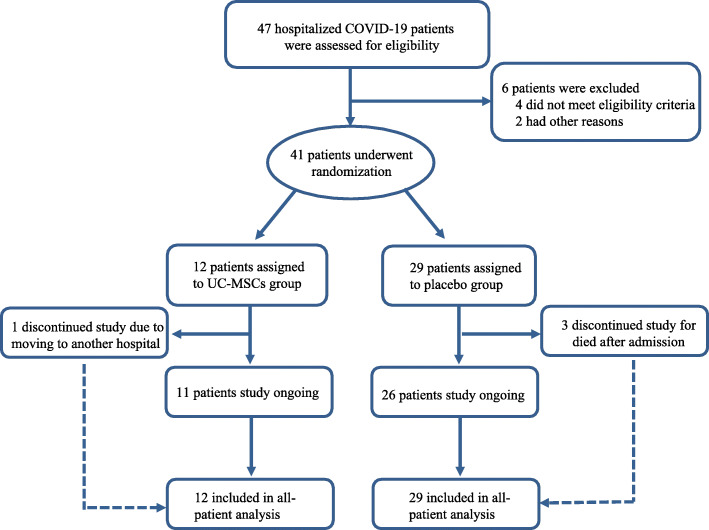
Flow diagram of the clinical trial in this study. Abbreviations: UC-MSCs, umbilical cord mesenchymal stem cells; COVID-19, coronavirus disease 2019

This study was conducted from February 12, 2020, to March 25, 2020. A total of 12 patients were enrolled in the hUC-MSC treatment group, and 29 patients were enrolled in the control group (Fig. 1). The median age of the patients was 61 years old (interquartile range [IQR], 50 to 70.5 years, with a median of 65 years in the hUC-MSC group vs. 58 years in the control group, *P* = 0.576), and 58.54% of the total patients were men (66.67% in the hUC-MSC group vs. 55.17% in the control group, *P* = 0.794, Table 1). The median time from onset of symptoms to enrollment was 13 days (11.5 days in the hUC-MSC group vs. 14 days in the control group, *P* = 0.135, Table 2). There were no significant differences between the hUC-MSC treatment and control groups in terms of demographic characteristics, laboratory test results, distribution of sequential scale scores, or NEWS2 scores at enrollment. In the trial, all patients used antiviral drugs for 7 to 10 days, and systemic glucocorticoids were also used for a median of 5 days (7.5 days in the hUC-MSC group vs. 5 days in the control group, *P* = 0.195, Table 2).

**Table 1:** Demographics and baseline characteristics of all patients

Variables	Total patients (***n*** = 41)	hUC-MSC(***N*** = 12)	Control (***N*** = 29)	***P*** value
Age (mean ± SD)	58.78 ± 16.26	61.00 ± 17.87	57.86 ± 15.79	0.576
Male sex, *n* (%)	24 (58.54%)	8 (66.67%)	16 (55.17%)	0.794
**Comorbidities,** *n* (%)				
Diabetes	8 (19.51%)	3 (25%)	5 (17.24%)	0.672
Hypertension	9 (21.95%)	3 (33.33%)	6 (20.69%)	1.000
**Signs and symptoms on admission**, *n* (%)				
Fever ≥ 37.3 °C	36 (87.80%)	10 (83.33%)	26 (89.66%)	0.470
Cough	27 (65.85%)	8 (66.67%)	19 (65.52%)	1.000
Respiratory rate > 24/min	31 (75.61%)	11 (91.67%)	20 (68.97%)	0.231
**Routine bloodwork**				
WBC count (× 10^9^/L) mean (IQR)	6.88 (5.06, 9.20)	7.37 (5.06, 11.16)	6.88 (5.06, 8.71)	0.449
3.5~9.5 (× 10^9^/L), *n* (%)	29 (70.73%)	7 (58.34%)	22 (75.86%)	0.068
< 3.5 (× 10^9^/L), *n* (%)	3 (7.31%)	1 (8.33%)	2 (6.89%)	1.000
> 9.5 (× 10^9^/L), *n* (%)	7 (17.07%)	4 (33.33%)	3 (10.34%)	0.165
LYM count (× 10^9^/L) median (IQR)	0.82 (0.59, 1.19)	0.77 (0.43, 1.72)	0.82 (0.59, 1.11)	0.783
< 1(× 10^9^/L), *n* (%)	18 (43.90%)	5 (41.67%)	13 (44.83%)	1.000
≥ 1(× 10^9^/L), *n* (%)	23 (56.10%)	7 (58.33%)	16 (55.17%)	1.000
MON count (× 10^9^/L) median (IQR)	0.50 (0.32, 0.75)	0.41 (0.26, 0.65)	0.62 (0.33, 0.0.91)	0.187
Hb (g/L) mean (IQR)	120.0 (112, 128.5)	119.5 (100.3, 127.8)	120.0 (112.0, 133.0)	0.322
PLT count (× 10^9^/L) median (IQR)	205.0 (145.0, 242.0)	207.0 (170.5, 327.8)	205.0 (141.0, 236.0)	0.338
< 100 (× 10^9^/L), *n* (%)	3 (7.32%)	1 (8.33%)	2 (6.90%)	1.000
≥ 100 (× 10^9^/L), *n* (%)	38 (92.68%)	11 (91.67%)	27 (93.10%)	1.000
PT (s) median (IQR)	12.30 (11.60, 13.20)	11.85 (11.33, 13.10)	12.40 (11.80, 13.20)	0.296
APTT (s) median (IQR)	37.10 (32.53, 41.80)	34.45 (29.60, 43.45)	38.80 (34.00, 41.80)	0.317
D-D (μg/L) median (IQR)	0.54 (0.02, 0.54)	0.89 (0.24, 2.78)	0.34 (0.20, 1.34)	0.224
**Myocardial enzymes**				
CK (U/L) median (IQR)	109.0 (40.5, 215.0)	162.5 (75.0, 360.8)	106.0 (36.0, 201.0)	0.132
< 310 (U/L), *n* (%)	36 (87.80%)	9 (75.00%)	27 (93.10%)	0.139
≥ 310 (U/L), *n* (%)	5 (12.20%)	3 (25.00%)	2 (6.90%)	0.139
LDH (U/L) median (IQR)	331.0 (229.0, 410.5)	285.5 (220.0, 392.0)	331.0 (237.5, 441.0)	0.338
< 250 (U/L), *n* (%)	14 (34.15%)	4 (33.33%)	10 (34.48%)	1.000
≥ 250 (U/L), *n* (%)	27 (65.85%)	8 (66.67%)	19 (65.52%)	1.000
**Biochemical indicators**				
ALT (U/L) median (IQR)	56.00 (42.50, 74.50)	67.00 (47.50, 104.00)	42.50 (42.00, 74.50)	0.065
< 50 (U/L), *n* (%)	9 (21.95%)	2 (16.67%)	7 (24.14%)	0.702
≥ 50 (U/L), *n* (%)	32 (78.05%)	10 (83.33%)	22 (75.86%)	0.702
AST (U/L) median (IQR)	31.00 (24.50, 38.00)	34.00 (25.25, 45.00)	31.00 (24.00, 37.50)	0.576
< 40 (U/L), *n* (%)	32 (78.05%)	8 (66.67%)	24 (82.76%)	0.407
≥ 40 (U/L), *n* (%)	9 (21.95%)	4 (33.33%)	5 (17.24%)	0.407
Cr (μmol/L) mean (SD)	60.27 (49.28, 69.46)	53.84 (44.97, 65.57)	65.85 (49.41, 71.59)	0.360
BUN (mmol/L) median (IQR)	5.05 (3.43, 6.15)	5.43 (3.14, 6.59)	4.83 (3.43, 5.90)	0.827

*IQR* interquartile range, *WBC* white blood cell, *NEU* neutrophil, *LYM* lymphocyte, *Mon* monocyte, *PLT* platelet, *Hb* hemoglobin, *PT* prothrombin time, *APTT* activated partial thromboplastin time, *D-D* DD dimers, *CK* creatine kinase, *LDH* lactate dehydrogenase, *ALT* alanine aminotransferase, *AST* aspartate aminotransferase, *Cr* creatinine, *BUN* urea nitrogen

**Table 2:** Patient status and treatments received at or after enrollment

Variables	Total patients (***n*** = 41)	hUC-MSC (***N*** = 12)	Control (***N*** = 29)	***P*** value
**NEWS2 score at day 1-median (IQR)**	8.00 (7.00, 10.00)	9.0 (8.00, 10.75)	8.00 (7.00, 10.00)	0.098
**Seven-category scale at day 1**				
3: Hospitalization, not requiring supplemental oxygen, No. (%)	3 (7.31%)	1 (8.33%)	2 (6.90%)	1.000
4: Hospitalization, requiring supplemental oxygen, No. (%)	28 (68.30%)	7 (58.33%)	21(72.41%)	0.469
5: Hospitalization, requiring HFNC or noninvasive mechanical ventilation, No. (%)	10 (24.39%)	4 (33.33%)	6 (20.69%)	0.441
6: Hospitalization, requiring ECMO, invasive mechanical ventilation, or both, No. (%)	0	0	0	1.000
Days from illness onset to randomization median (IQR)	13.00 (9.00, 17.50)	11.50 (6.00, 20.00)	14.00 (10.00, 18.00)	0.135
Earlier (≤ 12 days of symptom onset), No. (%)	17 (41.46%)	7 (58.33%)	10 (34.48%)	0.184
Later (> 12 days of symptom onset), No. (%)	24 (58.54%)	5 (41.67%)	19 (65.52%)	0.184
**Treatments during study period, No. (%)**				
Noninvasive mechanical ventilation	5 (12.20%)	3 (25%)	2 (6.70%)	0.139
Invasive mechanical ventilation	4 (9.76%)	0	4 (13.79%)	0.302
Antibiotic agent	36 (87.80%)	10 (83.33%)	26 (89.66%)	0.620
Antiviral treatment	41 (100%)	12 (100%)	29 (100%)	1.000
Vasopressors	7 (17.07%)	0	7 (24.14%)	0.085
Renal-replacement therapy	0	0	0	1.000
ECMO	0	0	0	1.000
Glucocorticoid therapy	41 (100%)	12 (100%)	29 (100%)	1.000
Days of glucocorticoid therapy-median (IQR)	5.00 (3.00, 8.50)	7.50 (5.00, 9.75)	5.00 (3.50, 9.00)	0.195

Notes: ECMO denotes extracorporeal membrane oxygenation, HFNC denotes high-flow nasal cannula for oxygen therapy, and NEWS2 denotes National Early Warning Score 2

### Primary outcome

In the hUC-MSC treatment group, all patients improved and were discharged, and no invasive ventilation occurred in 12 patients. The incidence of progression from severe to critical illness and the 28-day mortality rate were 0, while 4 patients in the control group deteriorated to critical illness and received invasive ventilation. Three of them died, and the 28-day mortality rate was 10.34%. The time to clinical improvement in the hUC-MSC treatment group was shorter than that in the control group (median, 9.0 days in the hUC-MSC group vs. 14.0 days in the control group, *P* = 0.006). In the age ≤ 65 years subgroup, the time to improvement in the hUC-MSC treatment group was 6.0 days (3.00, 7.00) vs. 12 days (7.25, 15.50) in the control group. In the age > 65 years subgroup, the time to clinical improvement was significantly prolonged in both groups: 13 days (11.75, 14.00) in the hUC-MSC treatment group vs. 23 days (18.50, 29.00) in the control group. Symptoms of weakness and fatigue, shortness of breath, and low oxygen saturation obviously improved in the hUC-MSC group compared with the control group. On day 3 of infusion, 2 patients (16.67%) in the hUC-MSC group had a reduction in the above symptoms; however, only 1 patient (3.45%) exhibited symptom relief in the control group. On day 7, more than half of the patients (58.33%) in the hUC-MSC group had symptom relief, 66.67% of patients did not require supplemental oxygen (Table 3); however, only 5 patients (17.24%) in the control group felt relief, and 3 patients (10.34%) did not receive oxygen supplementation. At day 14, 11 patients (91.67%) experienced obvious clinical symptom improvement in the hUC-MSC group, usually manifesting as significant remission of dyspnea and obvious absorption on imaging; however, only 15 patients (51.72%) felt symptom relief in the control group. All patients who received hUC-MSC treatment had no adverse reactions (such as rash, allergic reaction, and febrile reaction after infusion).

**Table 3:** Clinical outcomes of the patients

Variables	Total patients (***n*** = 41)	hUC-MSC (***N*** = 12)	Control (***N*** = 29)	***P*** value
**Time to clinical improvement-median, No. of days (IQR)**	13.00 (7.00, 18.50)	9.00 (6.00, 13.00)	14.00 (9.50, 21.00)	0.006
Age ≤ 65 years- median, No. of days (IQR)	9.50 (6.75, 14.00)	6.00 (3.00, 7.00)	12.00 (7.25, 15.50)	0.0014**
Age > 65 years –median, No. of days (IQR)	17.00 (13.00, 23.00)	13.00 (11.75, 14.00)	23.00 (18.50, 29.00)	< 0.001***
**Day 28 mortality, No. (%)**	3 (7.31%)	0	3 (7.31%)	0.543
Earlier (≤ 12 days after onset of symptoms)	0	0	0	
Later (> 12 days after onset of symptoms)	3 (7.31%)	0	3 (7.31%)	0.543
Patients who progressed from severe to critical illness, **No. (%)**	4 (9.76%)	0	4 (13.79%)	0.667
**Clinical improvement, No. (%)**				
Day 3	3 (7.32%)	2 (16.67%)	1 (3.45%)	0.200
Day 7	12 (29.27%)	7 (58.33%)	5 (17.24%)	0.020*
Day 14	26 (63.41%)	11 (91.67%)	15 (51.72%)	0.03
Day 28	37 (90.24%)	12 (100%)	25 (86.21%)	0.302
Hospital stay, median, No. of days (IQR)	22.00 (19.50, 25.00)	20.00 (16.00, 24.00)	24.00 (20.00, 26.50)	0.054
**Score on seven-category scale at day 7 of enrollment, No. of patients (%)**				
2: Not hospitalized but unable to resume normal activities	1 (2.44%)	1 (8.33%)	0	0.293
3: Hospitalization, not requiring supplemental oxygen	10 (24.39%)	7 (58.33%)	3 (10.34%)	0.0028**
4: Hospitalization, requiring supplemental oxygen	22 (53.66%)	3 (25.00%)	19 (65.52%)	0.037
5: Hospitalization, requiring HFNC or noninvasive mechanical ventilation	6 (14.63%)	1 (8.33%)	5 (17.24%)	0.651
6: Hospitalization, requiring ECMO, invasive mechanical ventilation, or both	2 (4.88%)	0	2 (4.88%)	1.000
7: Death	0	0	0	1.000
**Score on seven-category scale at day 14 of enrollment, No. of patients (%)**				
2: Not hospitalized but unable to resume normal activities	10 (24.39%)	5 (41.67%)	5 (17.24%)	0.124
3: Hospitalization, not requiring supplemental oxygen	23 (56.10%)	6 (50.00%)	17 (58.62%)	0.734
4: Hospitalization, requiring supplemental oxygen	2 (4.88%)	1 (8.33%)	1 (3.45%)	0.505
5: Hospitalization, requiring HFNC or noninvasive mechanical ventilation	2 (4.88%)	0	2 (6.70%)	1.000
6: Hospitalization, requiring ECMO, invasive mechanical ventilation, or both	1 (2.44%)	0	1 (3.45%)	1.000
7: Death	3 (7.32%)	0	3 (10.34%)	0.543

Note: Clinical improvement was defined as a decline of two categories on the modified seven-category ordinal scale of clinical status, or hospital discharge

*HFNC* high nasal flow oxygen therapy, *ECMO* extracorporeal membrane oxygenation

*P < 0.05, **P < 0.01, ***P < 0.001, compared with the control group

### The efficacy outcome

**Fig. 2:**
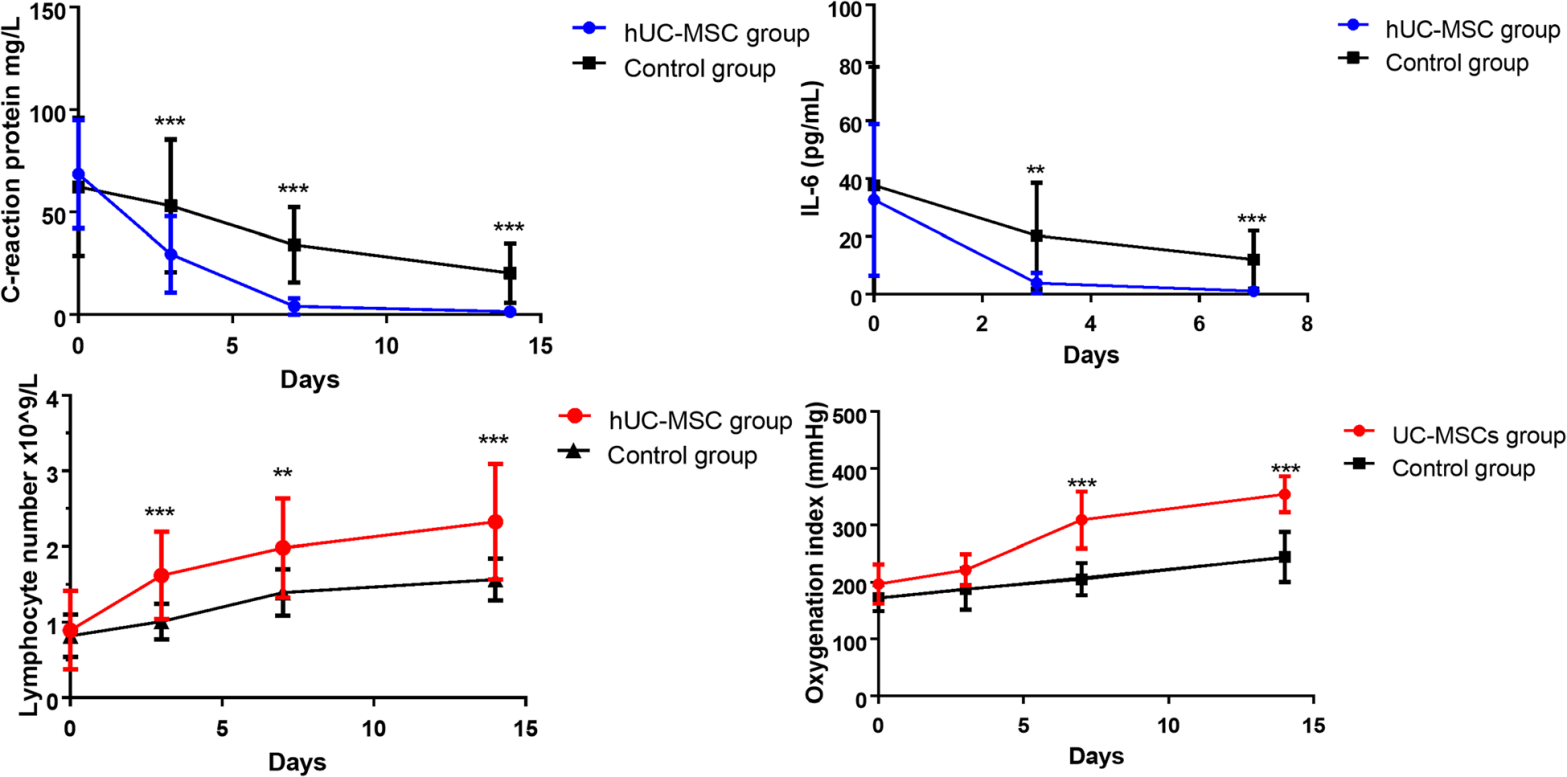
The dynamic changes in CRP, IL-6, oxygenation index, and lymphocyte number in patients in the hUC-MSC and control groups. (**P < 0.01, ***P < 0.001)

**Fig. 3:**
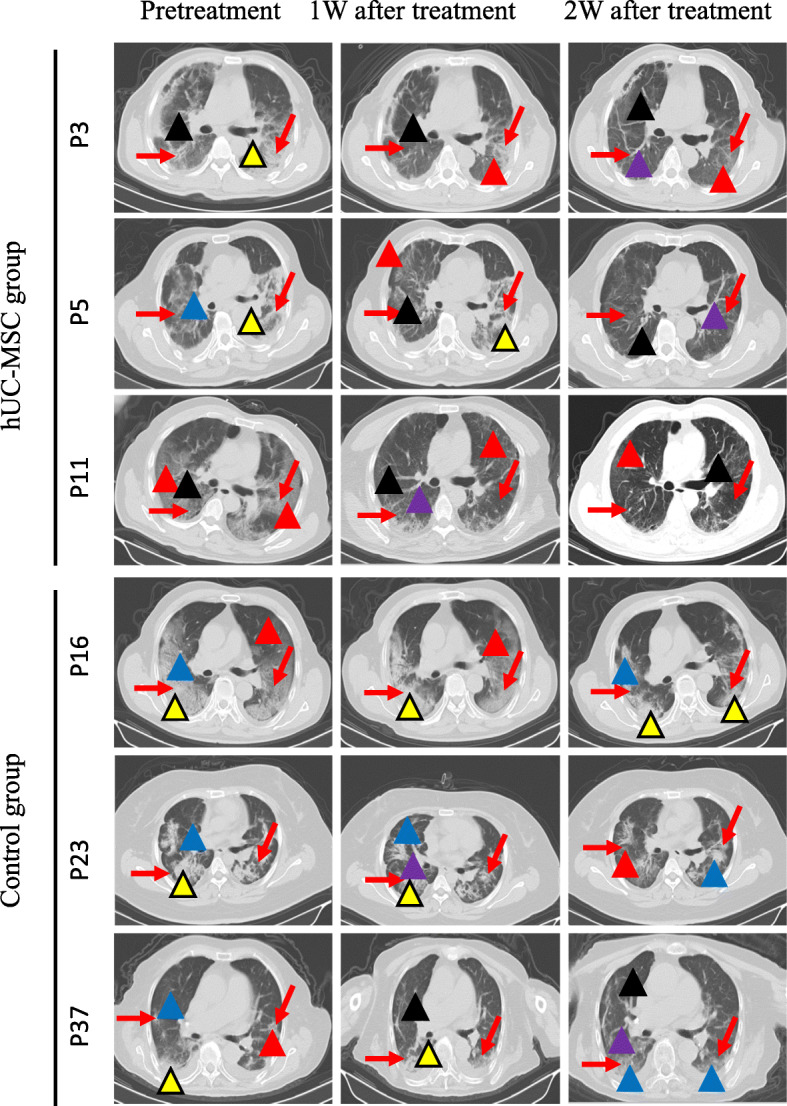
Chest computed tomography (CT) images of the patients in the hUC-MSC and control groups. CT imaging results for 6 patients (P3, P5, and P11 indicate patients 3, 5, and 11 from the hu-MSC group; P16, P23, and P37 indicate patients 16, 23, and 37 from the control group) at 3 time points (pretreatment, 1 week after treatment, and 2 weeks after treatment). The red arrows show the sites of inflammatory exudation, consolidation, or absorption. The red triangles show the sites of Crazy-paving pattern; the yellow triangles show the sites of consolidation; the blue triangles show the sites of GGO; the black triangles show the sites interlobular septal thickening; the purple triangles show the sites of bronchial wall thickening

The efficacy of hUC-MSC treatment was reflected by changes in indicators. Compared with those of the control group, C-reactive protein and IL-6 levels were significantly decreased from day 3 of stem cell infusion in the hUC-MSC group. Arterial blood gas analysis showed that the time for the oxygenation index to return to the normal range was faster in the hUC-MSC treatment group than in the control group. The difference was obviously significant starting from day 7 of hUC-MSC infusion, which was consistent with the time window for the patients’ clinical symptom relief. The time for the lymphocyte count to return to the normal range was significantly faster after stem cell infusion than after the control treatment (Fig. 2). Chest CT scans indicated that CT scores, the number of lobes involved, GGO, and consolidation, which reflected reduced lung inflammation in the stem cell treatment group, were significantly better than those in the control group (Fig. 3, Table 4 and Supplementary figure 1).

**Table 4:** Comparison of pretreatment and follow-up CT features

Parameters	Total patients (***n*** = 41)	hUC-MSC (***N*** = 12)	Control (***N*** = 29)	***P*** value
**Pretreatment**				
**CT score**	18.00 (15.00, 20.00)	18.50 (16.25, 20.75)	16.00 (15.00, 20.00)	0.1946
**Number of lobes involved**	4 (4, 5)	4 (4, 5)	4 (3.5, 5)	0.5826
**GGO**	41 (100%)	12 (100%)	29 (100%)	1.0000
**Linear opacities**	36 (87.80%)	10 (83.33%)	26 (89.66%)	0.6197
**Consolidation**	35 (85.37%)	11 (91.67%)	25 (86.21%)	1.0000
**Interlobular septal thickening**	35 (85.37%)	10 (83.33%)	25 (86.21%)	1.0000
**Crazy-paving pattern**	22 (53.65%)	7 (58.33%)	15 (51.72%)	0.7437
**Subpleural curvilinear line**	16 (39.02%)	6 (50.00%)	10 (34.48%)	0.7300
**Bronchial wall thickening**	27 (65.85%)	8 (66.67%)	19 (65.52%)	1.0000
**Lymph node enlargement**	20 (48.78%)	5 (41.67%)	15 (51.72%)	0.7337
**Pleural effusion**	5 (12.20%)	2 (16.67%)	3 (10.34%)	0.6197
**2 weeks after treatment**^**§**^				
**CT score**	9.00 (8.00, 10.50)	8.50 (7.25, 9.00)	10.00 (8.50, 12.50)	0.017*
**Number of lobes involved**	3 (2, 3)	2 (2, 2)	3 (2, 3)	< 0.001***
**GGO**	23 (58.97%)	4 (33.33%)	19 (70.37%)	0.0407*
**Linear opacities**	26 (66.67%)	5 (41.67%)	21 (77.78%)	0.0624
**Consolidation**	27 (69.23%)	4 (33.33%)	20 (74.07%)	0.0306*
**Interlobular septal thickening**	25 (64.10%)	5 (41.67%)	20 (74.07%)	0.0636
**Crazy-paving pattern**	16 (41.03%)	3 (25.00%)	13 (48.15%)	0.2913
**Subpleural curvilinear line**	12 (30.77%)	3 (25.00%)	9 (33.33%)	0.7190
**Bronchial wall thickening**	22 (56.41%)	4 (33.33%)	18 (66.67%)	0.0820
**Lymph node enlargement**	16 (41.03%)	3 (25.00%)	13 (48.15%)	0.2913
**Pleural effusion**	3 (7.69%)	1 (8.33%)	2 (7.41%)	1.0000

*GGO* ground-glass opacity

^§^During 2 weeks of treatment, two patients in the control group did not receive CT examination due to their serious illness, and the statistical number was calculated as 27 cases

*P < 0.05, **P < 0.01, ***P < 0.001, compared with control group

## Discussion

As the COVID-19 epidemic continues to spread and escalate, an increasing number of patients are being diagnosed with COVID-19 globally. However, to date, there are still no effective medical drugs or methods available, especially for the treatment of severe and critically ill patients.

At present, the mortality rate of COVID-19 varies due to the different sample size populations included in different regions and different severities of the epidemic [29, 30].

In our study, we found that no invasive ventilation occurred in 12 hUC-MSC-treated patients. The proportion of patients who progressed from severe to critical illness and the 28-day mortality rate were 0. In contrast, 4 patients in the control group progressed to critical illness and received invasive ventilation. Three of them died, and the 28-day mortality was 10.34%. Although the differences were not significant, the improvement trend is clear, and there is every reason to believe that it will be significant differences if the sample is large enough.

We also found that in the hUC-MSC treatment group, patients’ clinical symptoms, including chest tightness, shortness of breath, and fatigue, were significantly relieved and alleviated in a shorter time than observed in the control group. The levels of inflammatory factors, including IL-6 and CRP, could be rapidly reduced, and the lymphocyte count could return to normal levels in less time. As the patient’s chest tightness and shortness of breath quickly improved, arterial blood gas suggested that the oxygenation index could improve in a shorter time than observed in the control group. With the improvement of clinical symptoms, the changes in absorption on imaging were also obvious. The positive effect of hUC-MSCs on severe COVID-19 is clear, but the specific molecular mechanism of hUC-MSCs is not clear and still needs to be further illustrated.

MSC therapy can suppress excessive immune system activation and promote endogenous repair by improving the microenvironment. Studies have found that MSCs can enter the human body by intravenous infusion, and then some mesenchymal stem cells accumulate in the lungs, which can improve the lung microenvironment, protect alveolar epithelial cells, prevent pulmonary fibrosis, and improve lung function [31–33]. Based on previous studies and combined with our results, we speculate that hUC-MSCs can reduce the inflammatory response in the lungs by reducing the release of inflammatory factors mediated by immune regulation. Recently, several studies have reported that MSCs can regulate cell death [34–36], which occurs in lung disease, including ARDS, and may depend on paracrine factors and/or other modes of action, including gap junctions, tunneling nanotubes and extracellular vesicles [34]. However, whether MSCs inhibit cell death in COVID-19 requires further study.

In our study, in addition to the above results, we found another interesting phenomenon: patients with diabetes complications used significantly less exogenous insulin after hUC-MSC infusion than usual. The effects of hUC-MSCs on diabetes have been reported in many previous studies [37–39]. It has been reported that diabetes is a risk factor for death in COVID-19 patients [40–42], so for patients with severe COVID-19 with diabetes, hUC-MSC therapy may be the most ideal treatment. Previous studies indicated that older age is a potential risk factor for mortality in COVID-19 patients [40, 43]. In our study, patients younger than 65 years old had a good reaction to hUC-MSC therapy, which indirectly indicated the therapeutic effect of stem cells in patients with severe disease. The specific mechanism needs to be further clarified.

Because the researchers were unable to obtain sufficient stem cells at the time of urgent treatment, some patients who should have been randomized to the intervention group were assigned to the control group. In this study, we conducted sample randomization in the preliminary experimental design, which reduced the bias caused by enrollment to some extent.

The inherent challenges of conducting clinical trials in critically ill patients further expand the limitations of small sample sizes. In these patients, it is often difficult to discern whether a medical event is related to a potentially critical illness or the experimental therapy being tested. In our study, the requirement of baseline stability before MSC infusion was designed to reduce the noise of critical illness and make it more feasible to identify the potentially harmful effects of MSC infusion.

Our study found a therapeutic effect of hUC-MSCs on severe COVID-19. This is a single-center, small-sample controlled cohort study with limitations. First, the sample size was not large enough to stratify subgroups, and some bias was difficult to exclude. Second, this is a preliminary comparative clinical study, and the relevant mechanism needs to be further elucidated.

## Conclusions

As a noninvasive treatment, hUC-MSC therapy is a very effective and promising method for clinical application and promotion at the current critical moment due to the lack of effective approaches to treat severe COVID-19.

## Supplementary information

**Additional file 1: Supplementary Figure 1.** Chest computerized tomography (CT) images of the patients in hUC-MSC and control groups. CT imaging results for 41 patients (P1 to P12 were the patients treated with hUC-MSCs, and P13 to P41 were patients from control group. During 2 weeks of treatment, two patients (P20 and P25) in the control group did not receive CT examination due to their serious illness, and used bedside chest radiographs instead of CT scan.

## Data Availability

The majority of the data generated or analyzed during this study are included in this article. Unpublished data are available from the corresponding author upon reasonable request.

## Abbreviations

COVID-19: Coronavirus disease 2019
hUC-MSCs: Human umbilical cord mesenchymal stem cells
CRP: C-reaction protein
IL-6: Interleukin 6
IQR: Interquartile range
WBC: White blood cell
NEU: Neutrophil
LYM: Lymphocyte
Mon: Monocyte
PLT: Platelet
Hb: Hemoglobin
PT: Prothrombin time
APTT: Activated partial thromboplastin time
D-D: DD dimers
CK: Creatine kinase
LDH: Lactate dehydrogenase
ALT: Alanine aminotransferase
AST: Aspartate aminotransferase
Cr: Creatinine
BUN: Urea nitrogen
ECMO: Extracorporeal membrane oxygenation
HFNC: High-flow nasal cannula for oxygen therapy
NEWS2: National Early Warning Score 2
GGO: Ground-glass opacity
