# Supplementary material for: Treatment of severe COVID-19 with human umbilical cord mesenchymal stem cells
Source: Stem Cell Res Ther. 2020 Aug 18;11:361. doi: 10.1186/s13287-020-01875-5 (PMC7432540; doi:10.1186/s13287-020-01875-5)
Supplement: Supplementary file 1 — Additional file 1: Supplementary Figure 1. Chest computerized tomography (CT) images of the patients in hUC-MSC and control groups. CT imaging results for 41 patients (P1 to P12 were the patients treated with hUC-MSCs, and P13 to P41 were patients from control group. During 2 weeks of treatment, two patients (P20 and P25) in the control group did not receive CT examination due to their serious illness, and used bedside chest radiographs instead of CT scan. [file 13287_2020_1875_MOESM1_ESM.pptx]

## Slide 1
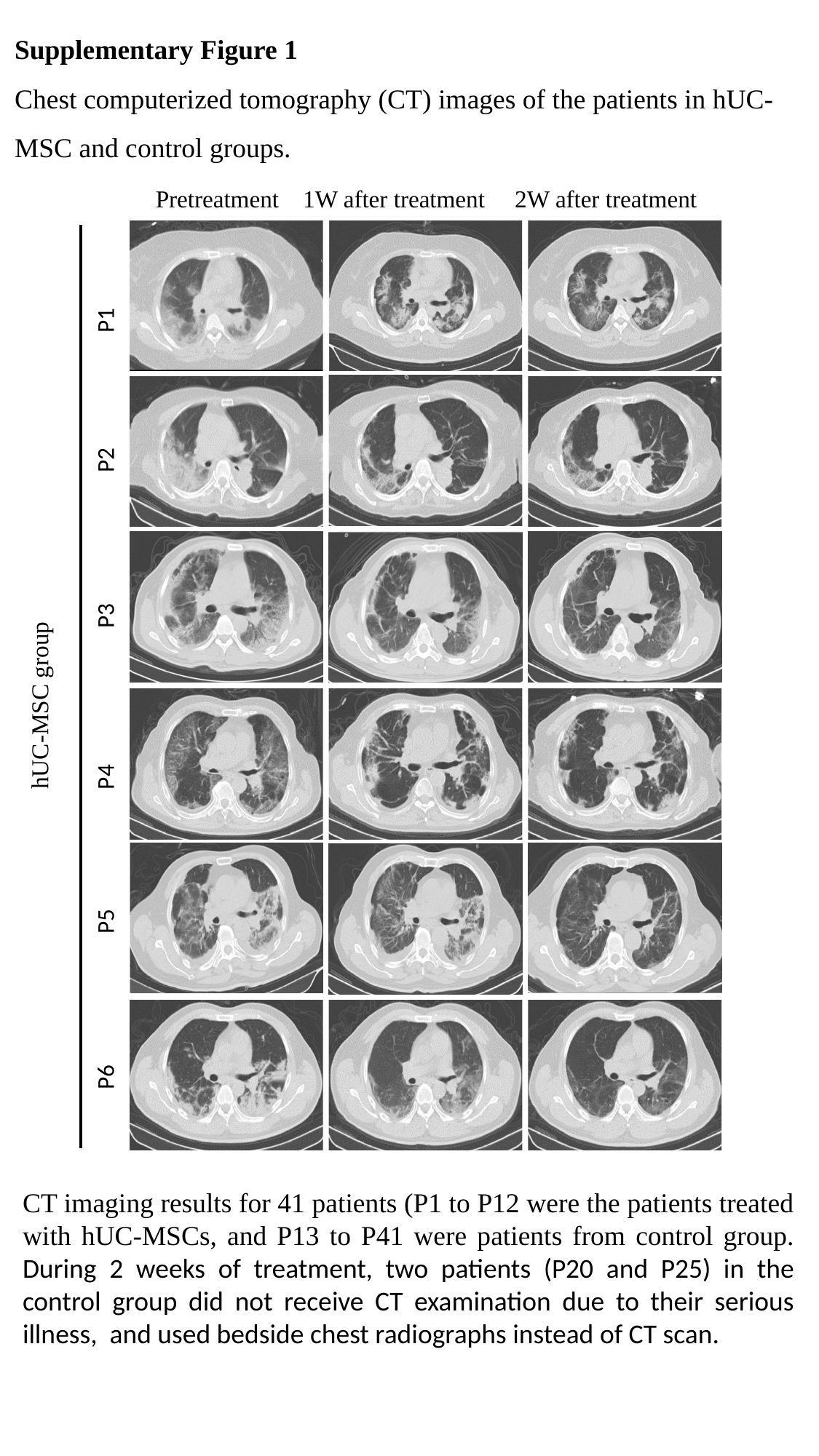

Supplementary Figure 1
Chest computerized tomography (CT) images of the patients in hUC-MSC and control groups.
Pretreatment 1W after treatment 2W after treatment
Pro-treated 1W after treated 2W after treated
P6 P5 P4 P3 P2 P1
hUC-MSC group
CT imaging results for 41 patients (P1 to P12 were the patients treated with hUC-MSCs, and P13 to P41 were patients from control group. During 2 weeks of treatment, two patients (P20 and P25) in the control group did not receive CT examination due to their serious illness, and used bedside chest radiographs instead of CT scan.

## Slide 2
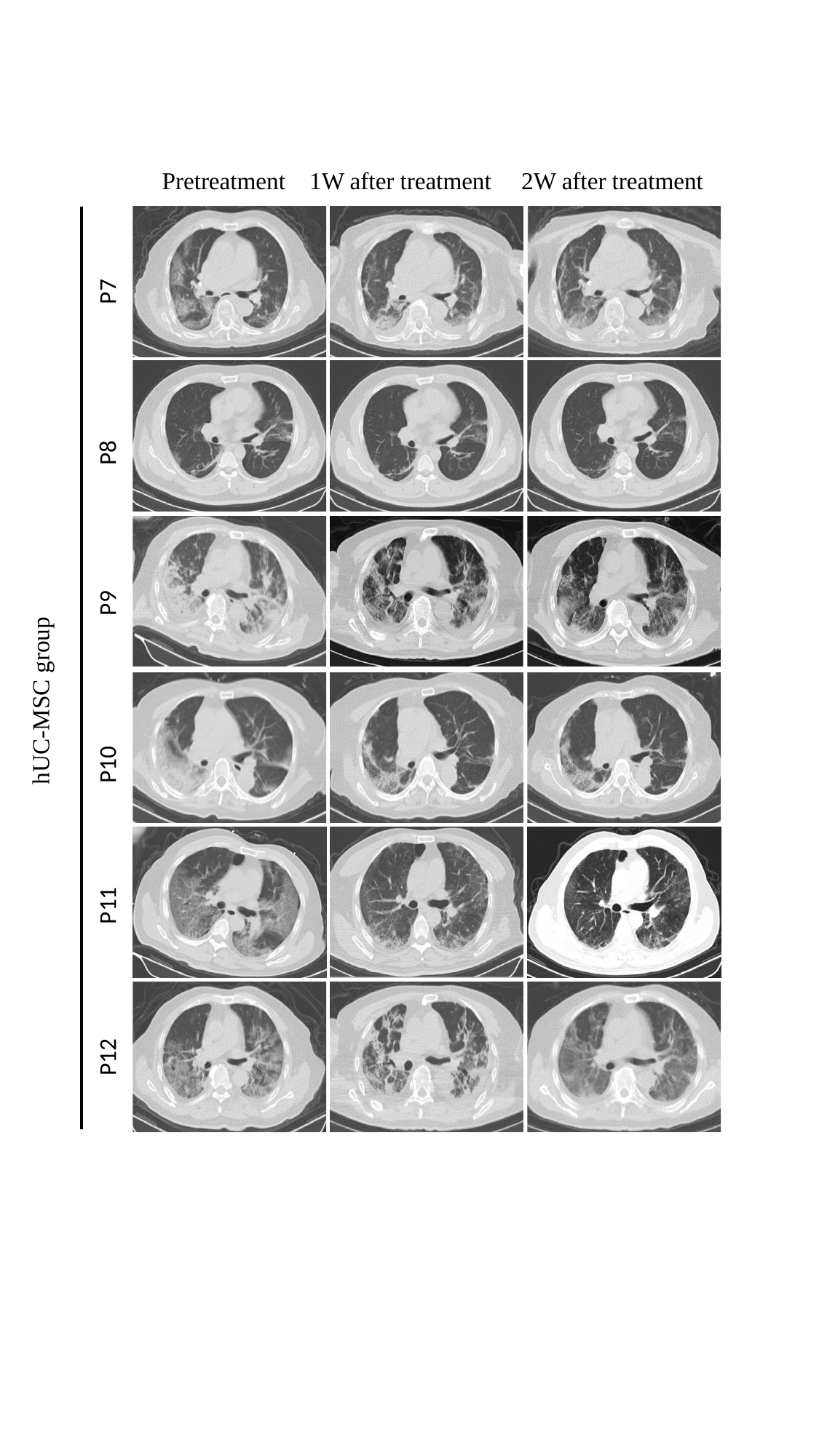

Pretreatment 1W after treatment 2W after treatment
 P12 P11 P10 P9 P8 P7
hUC-MSC group

## Slide 3
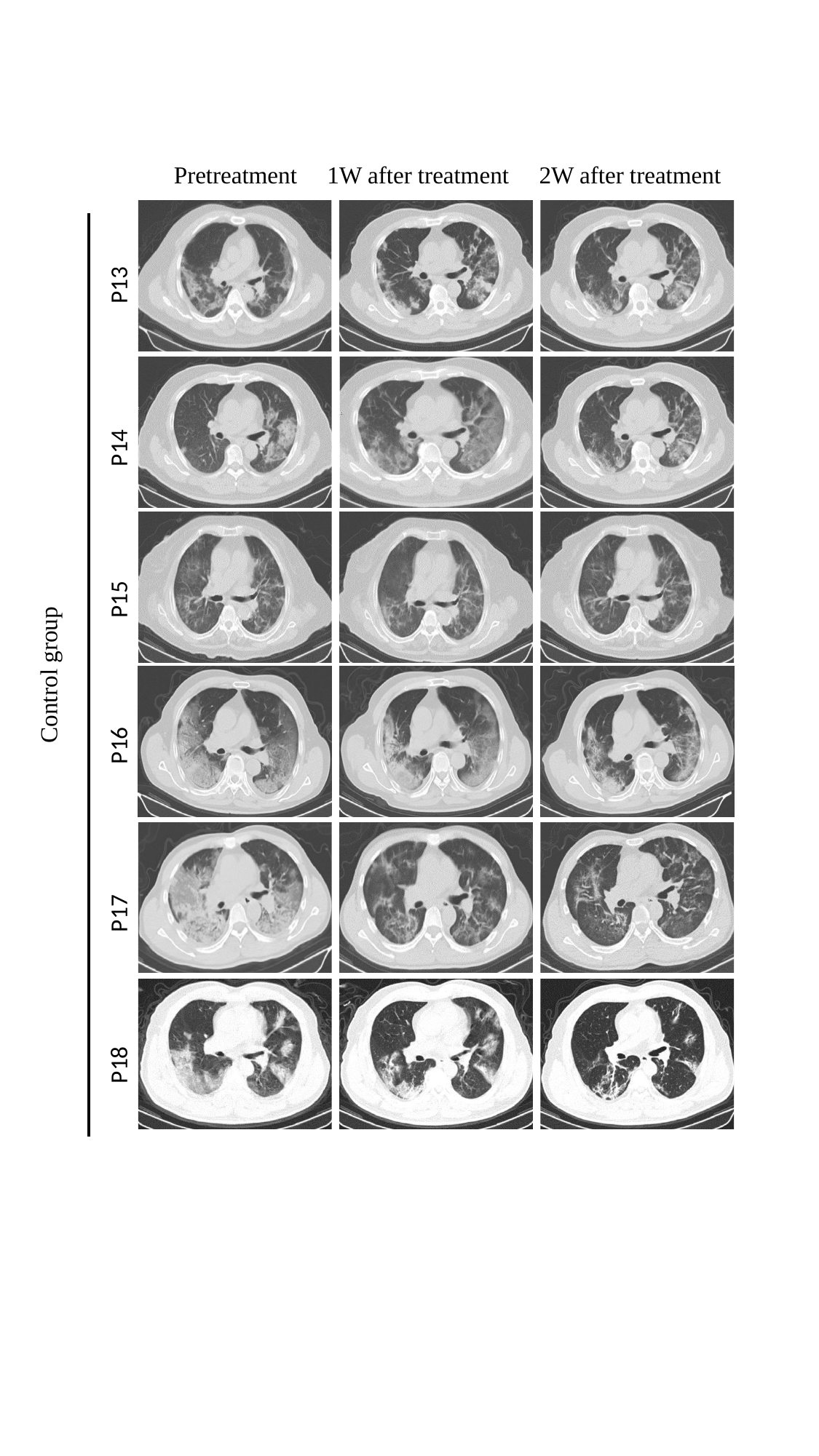

Pretreatment 1W after treatment 2W after treatment
P18 P17 P16 P15 P14 P13
Control group

## Slide 4
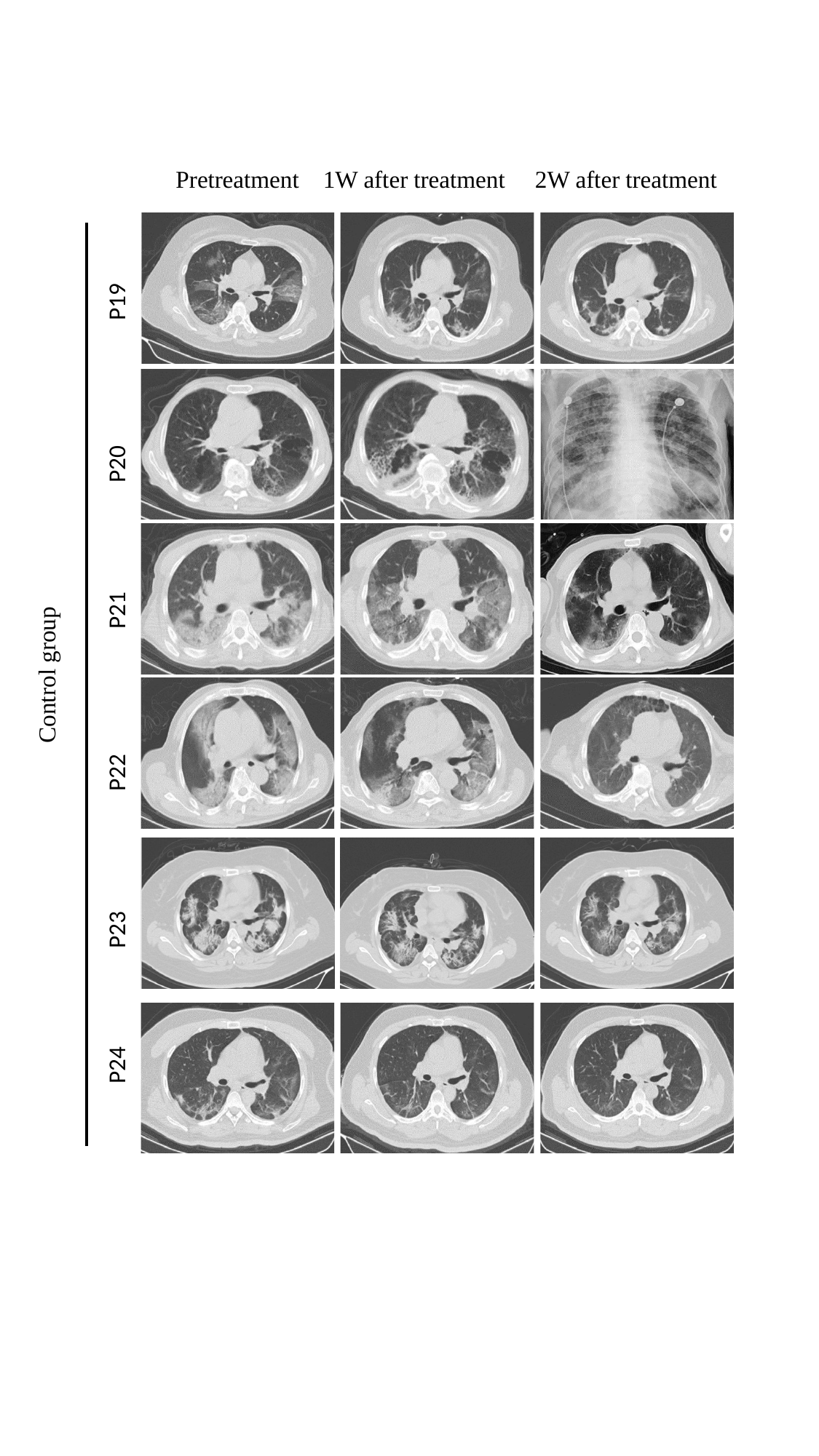

Pretreatment 1W after treatment 2W after treatment
P24 P23 P22 P21 P20 P19
Control group

## Slide 5
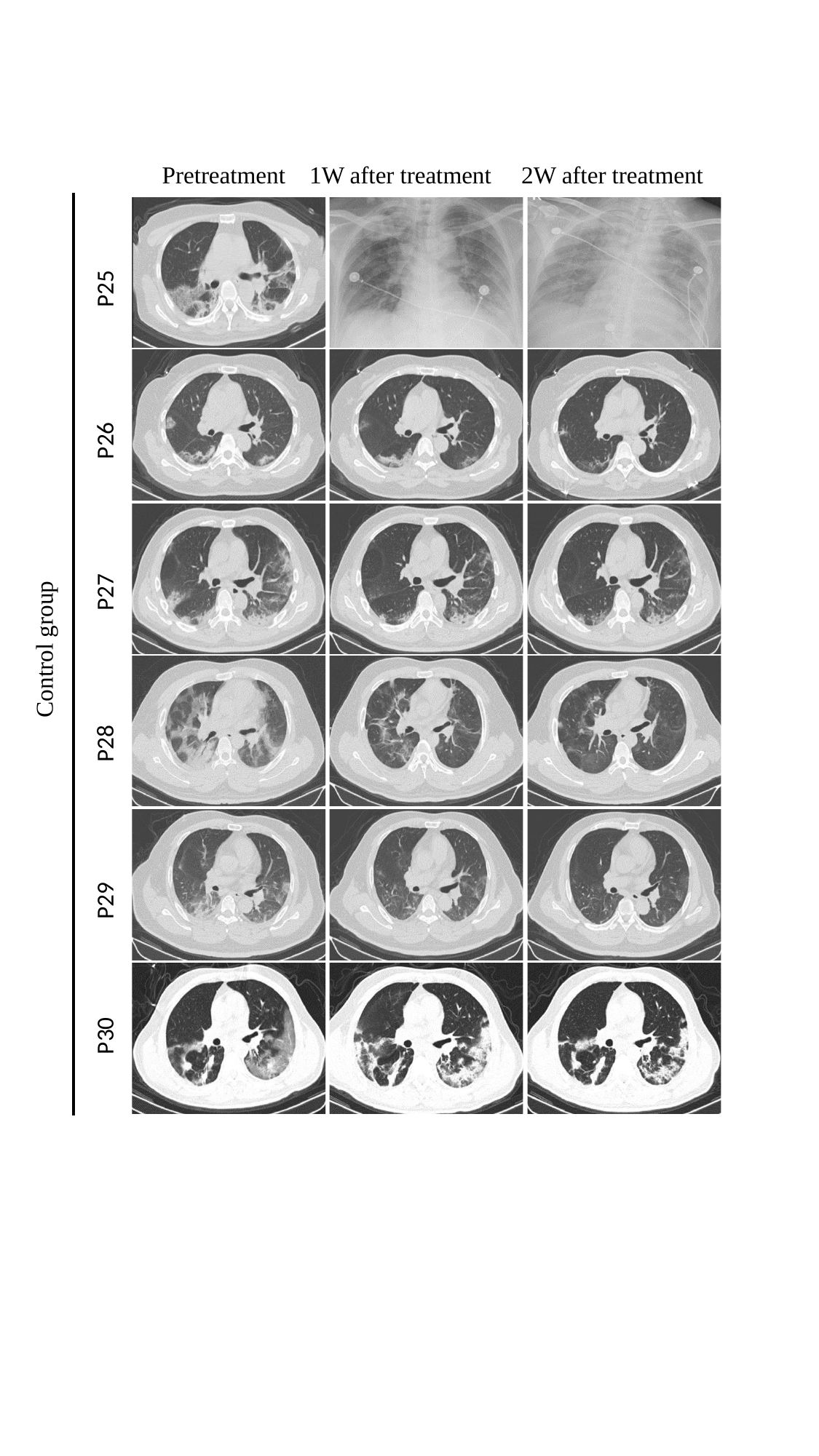

P30 P29 P28 P27 P26 P25
Control group
Pretreatment 1W after treatment 2W after treatment

## Slide 6
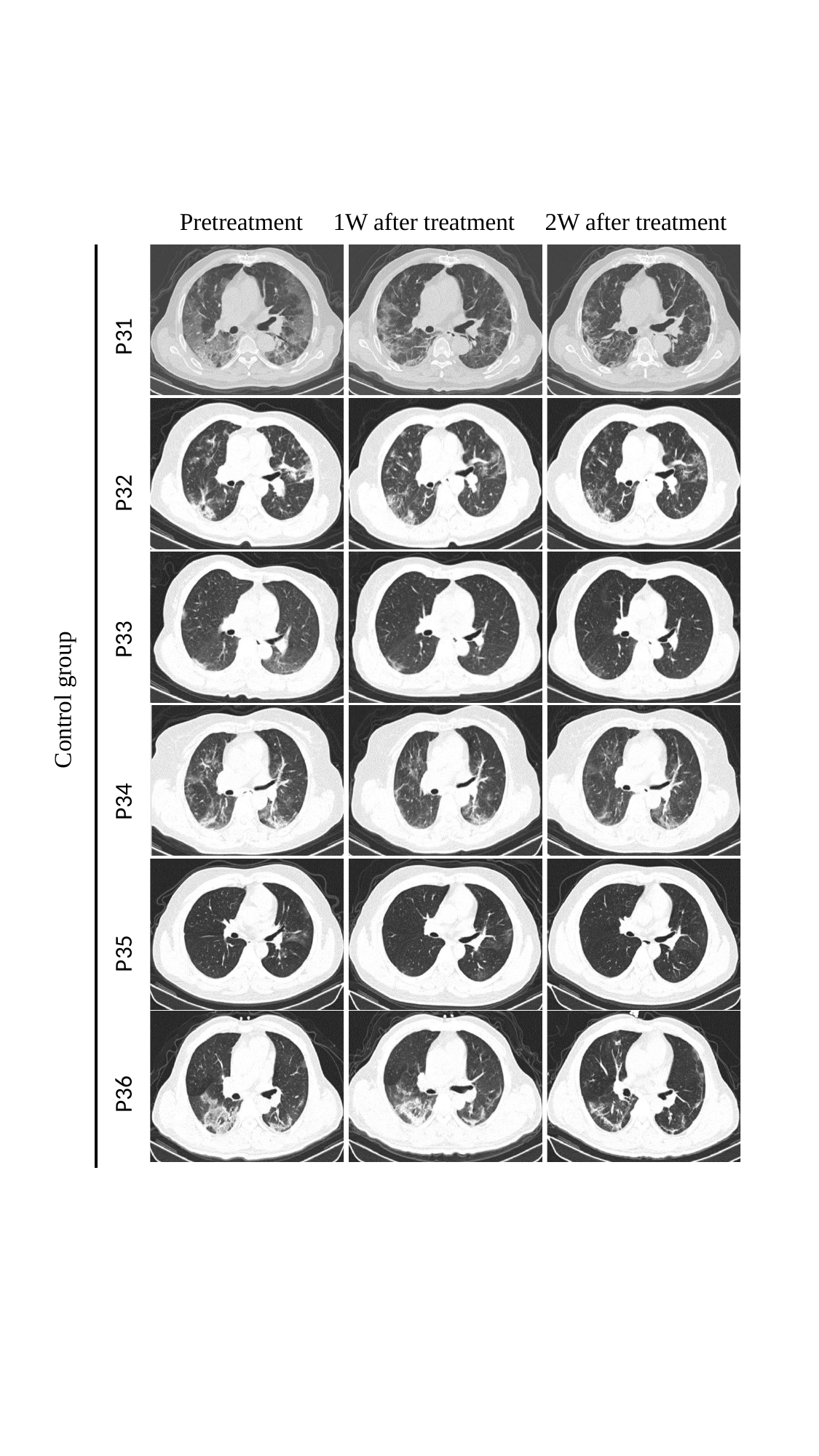

Pretreatment 1W after treatment 2W after treatment
Control group
 P36 P35 P34 P33 P32 P31

## Slide 7
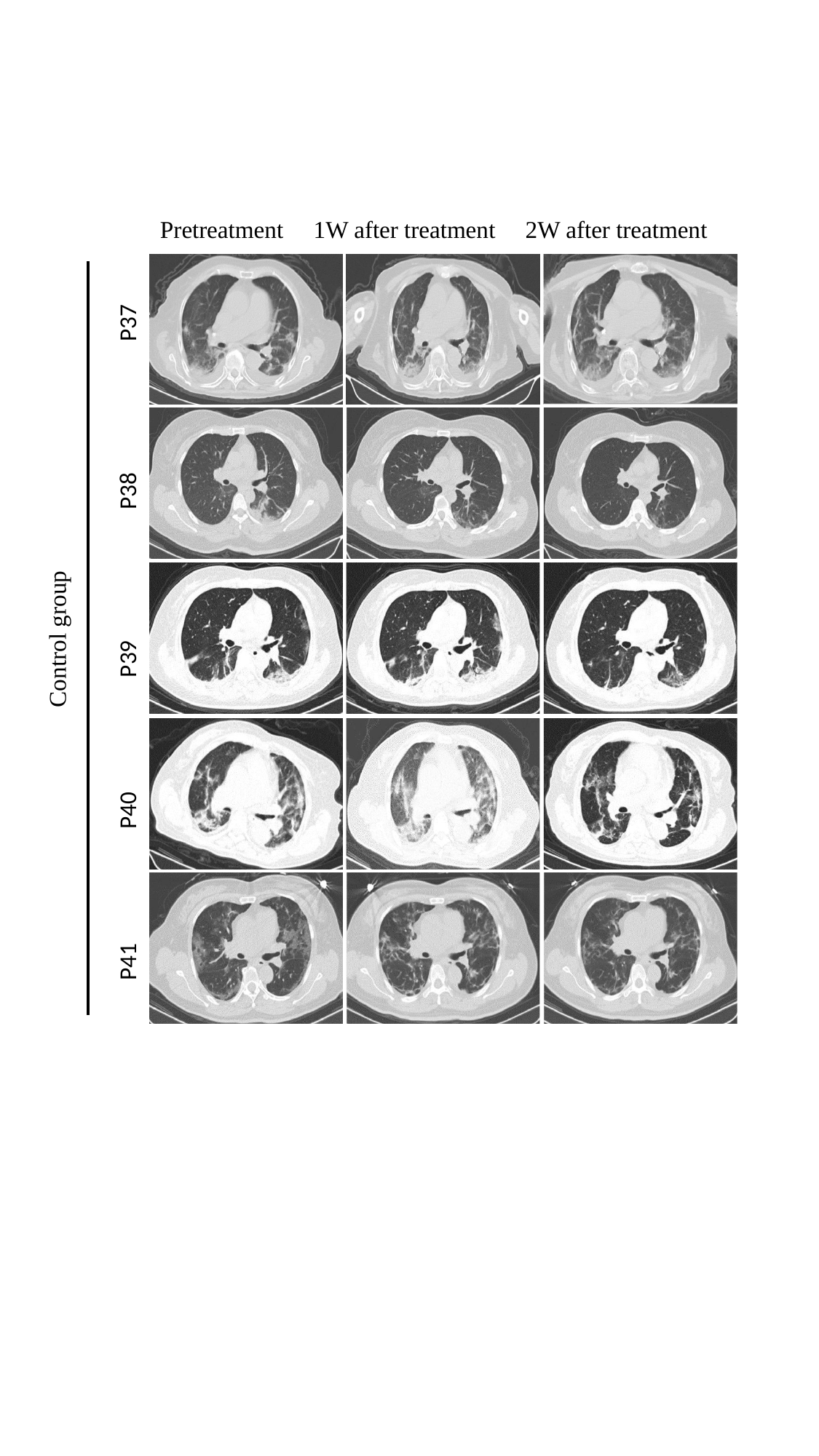

Pretreatment 1W after treatment 2W after treatment
 P41 P40 P39 P38 P37
Control group
